# Supplementary material for: Influenza A Virus Infection Reactivates Human Endogenous Retroviruses Associated with Modulation of Antiviral Immunity
Source: Viruses. 2022 Jul 21;14(7):1591. doi: 10.3390/v14071591 (PMC9320126; doi:10.3390/v14071591)
Supplement: Supplementary file 1 [file viruses-14-01591-s001.zip › MotifAnalysis.zip!/MotifOutput_SC35MdNS1/homerResults/motif1.similar.html]

motif1

## Information for motif1

A
C
G
T
G
A
T
C
A
C
G
T
C
G
T
A
A
C
T
G
A
G
T
C
A
C
G
T
A
G
T
C
C
G
T
A
C
T
G
A
A
C
T
G
A
C
T
G
  
Reverse Opposite:  

G
T
A
C
A
G
T
C
A
G
C
T
A
C
G
T
C
T
A
G
C
G
T
A
A
C
T
G
G
T
A
C
A
C
G
T
C
G
T
A
C
T
A
G
C
G
T
A
  

|  |  |
| --- | --- |
| p-value: | 1e-78 |
| log p-value: | -1.817e+02 |
| Information Content per bp: | 1.928 |
| Number of Target Sequences with motif | 39.0 |
| Percentage of Target Sequences with motif | 50.65% |
| Number of Background Sequences with motif | 116.9 |
| Percentage of Background Sequences with motif | 0.26% |
| Average Position of motif in Targets | 104.5 +/- 57.2bp |
| Average Position of motif in Background | 94.3 +/- 110.9bp |
| Strand Bias (log2 ratio + to - strand density) | 10.0 |
| Multiplicity (# of sites on avg that occur together) | 2.13 |
| Motif File: | file (matrix) reverse opposite |

### Similar de novo motifs found

|  |  |  |  |  |  |  |  |
| --- | --- | --- | --- | --- | --- | --- | --- |
| Rank | Match Score | Redundant Motif | P-value | log P-value | % of Targets | % of Background | Motif file |
| 1 | 0.748 | A C G T C T G A A C G T A G T C A C G T C G T A A C T G A G T C A C G T T A G C | 1e-74 | -170.702188 | 50.65% | 0.34% | motif file (matrix) |
| 2 | 0.832 | A G T C A C G T C G T A A C T G A G T C A C G T A G T C C T G A | 1e-57 | -133.390405 | 50.65% | 0.89% | motif file (matrix) |
| 3 | 0.706 | A C T G A C G T A G C T A C G T C G T A A C T G A G T C A C G T | 1e-48 | -111.072562 | 49.35% | 1.43% | motif file (matrix) |
| 4 | 0.624 | C G T A A C T G A G T C A C G T A T G C A G T C C T G A C T A G | 1e-6 | -14.478324 | 20.78% | 4.71% | motif file (matrix) |
